# A Comparative Study of Short-Term Social Media Use with Face-to-Face Interaction in Adolescence

**DOI:** 10.3390/children12040460

**Published:** 2025-04-03

**Authors:** Inês Mendonça, Franz Coelho, Belén Rando, Ana Maria Abreu

**Affiliations:** 1 Center for Interdisciplinary Research in Health (CIIS), Universidade Católica Portuguesa, 1649-023 Lisbon, Portugal; s-imsmendonca@ucp.pt (I.M.); s-fgcoelho@ucp.pt (F.C.); 2 Faculty of Health Sciences and Nursing (FCSE), Universidade Católica Portuguesa, 1649-023 Lisbon, Portugal; 3 Centre for Public Administration and Public Policies (CAPP), Institute of Social and Political Sciences, Universidade de Lisboa, 1300-663 Lisbon, Portugal; mcalvo@iscsp.ulisboa.pt; 4 Forward College, 1200-016 Lisbon, Portugal

**Keywords:** social media, ICT, smartphones, screen time, mental health, cognition, adolescents, digital culture

## Abstract

Background/Objectives: Previous research suggests that social media use can have immediate cognitive effects, raising concerns about its impact on adolescent cognition. This study aimed to examine the short-term cognitive effects of acute social media exposure and screen time habits by comparing cognitive performance in adolescents (13–15 years old) following 30 min of social media interaction versus face-to-face conversation, according to their screen time habits (more or less time spent in front of a screen). Methods: A total of 66 participants were divided into four groups: a social media group who used to spend less than 540 min per week in front of a screen (*n* = 19, a social media group with a habit of more than 540 min per week of screen time (*n* = 14), a face-to-face conversation group with a habit of less screen time per week (*n* = 15), and a face-to-face conversation group who used to spend more time per week in front of a screen (*n* = 18). Cognitive performance was assessed through attention (D2 Test), working memory (Corsi Blocks), abstract reasoning (Abstract Reasoning Test Battery), and inhibitory control (Go/No-Go Task). Additionally, mental effort was measured using a Visual Analogue Scale. Results: Contrary to our hypothesis, no significant differences emerged between groups in any cognitive domain or mental effort, with interaction modality and screen time showing no impact on response variables. Also, we found no significant interaction effect between factors. This suggests that a single 30-min session of social media use does not immediately impair cognition, nor does face-to-face interaction enhance it, despite screen time spent per week (when it varies from 135 to 540 min and from more than 540 to 1320 min). Conclusions: The absence of cognitive effects may be explained by excessive screen time as a key factor in cognitive impact and by the cultural integration of social media, creating a “ceiling effect” that minimizes the impact of short-term exposure and resembles addictive behavior. These findings emphasize the importance of a holistic approach involving families, schools, and governments to address both acute and cumulative social media use in adolescents.

## 1. Introduction

Smartphones are extensively used to bridge social distance through internet access, particularly via social media, with adolescents and young adults being the primary users [1]. Neuroplasticity plays a crucial role during childhood and adolescence, as the brain remains highly adaptable to learning and environmental stimuli at this stage [2]. The extensive cognitive and socio-affective development in adolescence involves significant brain changes. These include increased white matter connections that improve communication between regions and are linked to better behavioral control, such as an enhanced ability to delay gratification through the maturation of connections between the prefrontal cortex and subcortical striatum [3]. This critical period for neurodevelopment [4] raises concerns about the extensive use of smartphones. A Swedish study found that high smartphone screen time (defined as more than 4 h daily) increases the odds of having mental health problems in adolescents compared to minimal usage, while TV and computer screen time showed no significant impact, suggesting that smartphones, with their capacity for constant content consumption, may have a greater negative impact on mental health [5]. Also, a U.S. study with a sample of 17,076 adolescents found that those with excessive screen time were 1.28 times more likely to report serious cognitive difficulties than those with moderate use [6]. It is important to highlight that correlation does not imply causation [7], but these associations between social media and cognitive issues are a contemporary topic that raises significant concerns, justifying further investigation and motivating this study.

The use of smartphones has become a relevant topic, investigated for its benefits, such as improved work performance and learning [8,9], cognition [10,11], and communication with friends or family anytime [12,13,14]. However, the benefits of smartphones are debated, with evidence suggesting their regular use may negatively affect cognitive abilities such as memory, attention, and emotional regulation [11,15]. Excessive phone use and constant social media interaction are linked to cognitive and emotional impairments [16,17]. American youth spend an average of 7.5 h per day with media, more than any other waking activity, with 29% of that time spent multitasking across multiple media streams, a behavior associated with the following cognitive impacts: poorer memory, psychosocial effects like increased impulsivity, and neural structural changes, including reduced volume in the anterior cingulate cortex, a region involved in cognitive and social-emotional control [18]. Research also highlights the negative effects of compulsive use, especially among youth, with consequences for academic, social, and physical performance [8,19] and personal relationships [13,20].

Fear of missing out (FoMO), smartphone dependency, and the need to always have the phone present (Entrapment) impact cognitive performance [17,21]. FoMO affects many who feel compelled to participate in all social events out of fear of missing out on interactions, opinions, and significance, which fuels smartphone dependency driven by the expectation of prompt responses, leading to feelings of entrapment when responses are delayed, potentially harming relationships [21]. Also, symptoms of anxiety, agitation, tachycardia, and disorientation are reported in those suffering from Nomophobia (i.e., the fear of being without a mobile phone), who prefer virtual interactions, keep their phones close while sleeping, and often experience “ringxiety”, the false sensation of hearing notifications [22]. Nomophobia can disrupt sleep and cause anxiety and stress, especially among high school and university students [21,23]. The presence of a phone during a brief interaction reduces conversation quality [24].

Adolescents use smartphones for a variety of activities, including playing video games, engaging with social media, and watching video clips on platforms like YouTube [25]. However, beyond those activities, social media have raised significant concerns, particularly due to their association with poor adolescent mental health [26], their relationship with worsening academic performance [27], and their substantial role in adolescents’ daily screen time [28]. In 2018, about one-third of adolescents in Europe and North America were using social media almost continuously throughout the day [29], a figure that likely increased during the COVID-19 pandemic [30,31]. Whether users are giving or receiving “likes” (i.e., a feature on social media, functioning as both a monetary and social reward), brain regions involved in reward processing are activated, such as the striatum and ventral tegmental area [32], regions also related to addiction-like behaviors [33]. Social media algorithms are meticulously crafted to maximize user interaction, with interfaces designed to capture attention through mechanisms akin to slot machines, employing intermittent rewards that trigger dopamine release—related to motivation and desire—and reinforce engagement behaviors, while emotionally triggering stimuli further amplify online distraction [34,35]. Marketing techniques in social media, measured through electroencephalogram (EEG) and event-related potential (ERP), show that online content activates bilateral temporal brain areas in just milliseconds, rapidly capturing attention and fostering repeated behaviors by engaging users instantly [36], highlighting how social media engagement often revolves around brief moments that can reinforce habitual use.

The shift from brief adaptive to problematic social media use results from interactions between individual traits and cognitive-emotional responses, where habitual use becomes less rewarding and more compensatory, weakening inhibitory control and reinforcing compulsive behaviors by altering activity in executive control, reward, salience, and habit networks, affecting regions like the ventral striatum, amygdala, prefrontal cortex, anterior cingulate, and insula [37]. Social media interaction may stem from impulse-driven behaviors like habitual smartphone checking, notification distractions, or guilt over unread messages or inactivity, as well as from seeking rewards or escaping stressful situations [38]. Social media use often occurs in brief moments of distractions that repeatedly disrupt daily activities, potentially leading to cognitive effects like impulsivity and socio-emotional consequences such as stress [39]. A study found that after the acute social stress, those using Facebook immediately had higher sustained cortisol levels than those who read quietly, suggesting acute social media use may hinder stress recovery [40], which may impact executive functions [41]. Another study on acute social media use, body dissatisfaction, and eating behavior found that brief exposure disrupts body image through social comparison and reduces snack intake in females favoring thin ideals [42], potentially affecting reasoning by influencing the theory of mind and perceptual inferences on self and others’ images [43,44].

Previous studies have shown that having a phone present during face-to-face conversations disrupts human connection, reducing intimacy and hindering the development of closeness and trust [45]. Moreover, partner phone use in interactions can have a detrimental effect on relationship satisfaction [46]. However, less is known about the cognitive effect of mobile phone presence. Indeed, a study showed that the mere presence of our phones affects our cognitive capacity and that the cognitive costs associated with the presence of our phones were more substantial for those who are more dependent [14]. In this study, participants were requested to undergo cognitive tasks with their phones on their desks, in their pockets/bags, or in another room. The findings showed that the mere proximity of the phone taxed the participants’ attentional resources involved in fluid intelligence and working memory. Specifically, and from a cognitive perspective, social media use has been linked to poorer attentional control, as platforms like Facebook and Instagram promote constant distraction through notifications and updates, resulting in a state of continuous partial attention [47]. Media-induced stress impairs information processing, with high-arousal content diminishing the ability to concentrate after media use, thereby affecting attentional resources [48]. Regarding working memory, media multitaskers (i.e., using social media, such as Facebook, while watching television) may show lower working memory performance, as their broader attentional scope and higher impulsivity allow goal-irrelevant information to interfere with goal-relevant information [49,50]. Internet searches may impair brain activity related to memory [51], while social media influences collective memory, including the formation of false memories, such as fake news—a modern digital challenge—by serving as a space where information and misinformation coexist, often with sources that can be subtly manipulated [52,53]. Also, individuals who browse Instagram during class may experience a reduced capacity to retain information compared to those who do not browse Instagram, as social media distractions can impair short-term memory recall of novel information in social situations [54]. Concerning reasoning, watching short videos is associated with decreased analytical thinking, as the act of swiping through video feeds, rather than the content itself, negatively affects users’ ability to engage in deeper, more reflective reasoning [55]. The negative effects of social media on attention and memory may impair reasoning, as these cognitive processes are fundamental to higher-level functions [56]. Within reasoning components, abstract reasoning is essential for higher-order cognitive abilities in childhood and adolescence [57], it has a crucial role in critical thinking and aligns with the theory of mind, enabling individuals to recognize both their own and others’ cognitive biases and limitations [58]. A stronger capacity for abstract reasoning is linked to a greater inclination toward critical thinking [59]. Social media can foster confirmation bias by allowing users to engage primarily with content that reinforces their existing beliefs, helping them avoid cognitive dissonance but potentially weakening critical thinking skills [60]. Furthermore, adolescents are often receptive to social media content, as their critical thinking can be influenced by parasocial relationships with influencers [61]. Lastly, inhibitory control, a core executive function that regulates and suppresses inappropriate behavior, is less efficient in excessive social media users, who struggle with resource allocation and exhibit greater difficulty in late-stage inhibitory control compared to non-excessive users [62]. Also, as FoMO induced by social media intensifies, inhibitory control declines, as it depletes cognitive resources during early conflict detection, leaving insufficient capacity for later stages of the inhibitory process, suggesting that FoMO weakens inhibitory control within the social media context [63,64].

General intelligence is a key aspect of cognitive development, encompassing various processes that evolve, with attention control, working memory, and reasoning playing a more prominent role during childhood [65]. Inhibitory control is also a fundamental executive function in childhood and adolescence for regulating impulsivity and minimizing externalizing behaviors [66,67]. In light of the increasing cognitive impact that smartphones can have during the crucial neurodevelopment period of adolescence [4], along with rising concerns about social media use among this age group [26,28], we aim to investigate how acute social media use influences adolescent cognition, focusing on attentional processes, working memory, reasoning, and inhibitory control, as these functions are fundamental to cognitive development in childhood and adolescence and appear to be affected by social media [47,49,50,56,63]. Furthermore, acute social media exposure impacts cognition through stress, distraction, and self-comparison [39,40,42], potentially affecting processes linked to problematic social media use such as impulsivity and executive functions (e.g., attention, working memory, and inhibitory control) [68], as well as higher-order cognitive functions such as reasoning, particularly in self-comparison and identity processes in digital contexts [44]. Since problematic social media behavior begins as adaptive but can become addictive [37], we aimed to assess whether brief social media exposure could impair cognitive domains linked to problematic use, including attention, working memory, inhibitory control, and reasoning.

Additionally, social media affects mental workload, as interruptions during other tasks increase cognitive effort, making it more challenging to switch between activities [69]. Social media use also imposes a higher cognitive load, potentially impairing the completion of daily cognitive tasks such as learning [70]. Also, individuals with excessive screen time are more likely to experience significant cognitive difficulties compared to those with moderate usage [6]. Alongside the mentioned cognitive domains, we also evaluated mental effort and screen time to assess their respective impacts on the results.

We conducted a pre-test-post-test control group design comparing pre- with post-cognitive scores of a group exposed to acute social media (intervention group—IG) with a group engaging in face-to-face interaction (control group—CG). We hypothesized that significant differences would arise between the groups, with the IG exhibiting lower scores in attention, working memory, reasoning, and inhibitory control, along with higher mental effort compared to the CG, with these effects being more pronounced in individuals with greater screen time.

## 2. Materials and Methods

The pre-test-post-test control group design aimed to evaluate cognitive performance—specifically attention, working memory, reasoning, and inhibitory control—before and after a brief interaction with either social media or face-to-face conversation in adolescents. Drawing on literature that highlights the immediate cognitive effects of social media discussed previously [48,54,55], the study involved two groups: the IG that spent 30 min interacting on social media in isolation, and the CG that engaged in 30 min of face-to-face conversation in pairs. The objective was to determine whether short-term social media exposure affects cognitive performance compared to in-person interaction. Previously, the Raven’s Progressive Matrices test was applied to obtain a measure of general intelligence (G Factor). Then, participants were matched according to their G Factor score, and to their age. They were randomly allocated to groups to balance these variables.

### 2.1. Participants

A priori power analysis was conducted using G*Power v.3.1.9.4 [71] to determine the required sample size for a *t*-test comparing two independent means. We assumed a large effect size (d = 0.80), a power of 0.80, an alpha level of 0.05, and a one-tailed test, which resulted in an estimated sample size of *n* = 42. To account for potential attrition, we recruited *n* = 66 participants. Following data collection, we introduced an additional factor into the analysis (Screen Time). Consequently, a post hoc power analysis was conducted using an F-test for ANOVA (fixed effects, special, main effects, and interactions). We assumed a large effect size (f = 0.40), a power of 0.80, an alpha level of 0.05, one degree of freedom in the numerator, four groups, and a total sample size of *n* = 66. This analysis yielded an achieved power of 0.89.

Participants were recruited from two Portuguese schools located in the center-southern region of the Greater Lisbon area. Approval was obtained from the schools’ administrations before the study started. Informed consent was secured from all participants, with guardians providing a signed consent form, and the participants voluntarily agreeing to participate by signing an assent form. The forms were sent to guardians and participant minors after a researcher presented the study procedure and held a Q&A session specifically for this purpose.

We recruited 66 participants aged 13 to 15 years, who had a social media account and were enrolled in the 8th grade, in the third cycle of basic education. They were distributed across two groups: IG (*n* = 33, 18 girls, 15 boys), who participated in the social media intervention, and CG (*n* = 33, 22 girls, 11 boys), who participated in the face-to-face intervention. This study received approval from the local Ethics Committee.

As described before, the age and Raven’s Progressive Matrices [72] score of the participants were controlled, as both age and intellectual capacity play a crucial role in adolescent development [73]. To ensure no confounding effect of age, a *t*-test was performed and results showed no significant differences between CG (M = 13.15, SD = 0.364) and IG (M = 13.38, SD = 0.660), t(47.956) = −1.683, *p* = 0.099.

Regarding Raven’s Progressive Matrices test [72] it was administered during an initial phase before the intervention. This test assesses non-verbal intellectual ability, specifically the subject’s capacity for abstract reasoning to deduce and induce relationships. It consists of 60 matrices organized into five series (A, B, C, D, E), of 12 matrices each, increasing in difficulty. In each matrix, participants were presented with an incomplete pattern and had to select the correct missing pattern piece from six options in the A, B, and C series and eight options in C, D, and E options to complete the design. Each matrix had only one correct answer, and responses were recorded on a designated answer sheet. The Test lasted from 30 to 40 min. Total score from 0 to 60, based on the number of correct responses. To ensure no confounding effect of this variable, a t-test for independent samples revealing no statistical differences between the groups (t(63) = 1.435, *p* = 0.156), (M_CG_ = 47.55, SD = 5.40; M_IG_ = 45.31, SD = 7.05).

### 2.2. Instruments and Measures

#### 2.2.1. Attention Measure—D2 Test

Given the attentional issues linked to social media use [47] and its critical role in cognitive development during childhood and adolescence [65], we assessed participants’ attention using the D2 test of attention [74,75]. This is a pencil and paper test that assesses essential cognitive processes necessary for success in complex tasks, including selective attention, concentration, attention processing speed, information processing accuracy, and performance-related qualitative aspects. The task involves crossing out all instances of the letter ‘d’ with exactly two marks across 14 test lines, with each line completed in 20 s, resulting in a total duration of 4 min and 40 s. We considered key performance indicators and considered them as the outputs in results, total efficiency score (TE-S)—i.e., the total number of characters minus errors—for attention control and inhibition, concentration index (CI) for focus consistency, variability index (VI) for task execution stability, and error percentage (E%) for meticulousness and performance quality.

#### 2.2.2. Working Memory Measure—Corsi Blocks

Considering the effects of social media on working memory [49,50] and the importance of this cognitive domain during early and mid-life stages of life [65], we chose to assess working memory using the Corsi Blocks task [76]. In this task, blocks are tapped in a specific sequence, which the participant must replicate in the same order. The sequence length increases progressively, and the test concludes when the participant fails to accurately reproduce two sequences of the same length. The test lasted approximately 5 min.

#### 2.2.3. Abstract Reasoning Measure—Reasoning Test Battery

Abstract reasoning, correlated with fluid intelligence and problem-solving in novel situations, is essential for higher-order cognitive abilities in childhood and adolescence [57]. Abstract reasoning helps individuals recognize their own and others’ cognitive biases and limitations [58], but social media can impact this skill by reinforcing existing beliefs with confirmation bias and promoting uncritical acceptance of content [60,61]. We chose a Reasoning Test Battery (BPR): *Bateria de Provas de Raciocínio* [77]—used to assess the abstract reasoning of students between the 5th to 12th grades. Here, we used the BPR 7/9 version, designed for students from the 7th to 9th grades (the 3rd cycle of elementary education). The BPR consists of five tests that evaluate different types of reasoning: abstract, verbal, numerical, mechanical, and spatial, that were validated for the Portuguese population [78]. Here we only applied the abstract reasoning test. In the abstract reasoning test, participants must identify an abstract visual transformation (e.g., the addition of a triangle) that occurs between a first and second figure and apply the same transformation to a third figure to determine the correct fourth figure outcome from five options (A, B, C, D, E). The test lasted about 5 min. Scoring was obtained using specialized software provided by the test publisher.

#### 2.2.4. Inhibitory Control Measure—Go/No-Go Task

Social media may negatively impact inhibitory control [63], which is a key executive function during early and mid-development, essential for regulating impulsivity and reducing the risk of mental health issues associated with internalizing symptoms [66,67]. To assess inhibitory control in this context, we chose the Go/No-Go Task, a commonly used measure in research on inhibitory control deficits related to social media use [63,79]. We used the Go/No-Go Task through the free online version of the Testable suite of academic tools [80] to evaluate this cognitive domain, measuring response inhibition to visual stimuli on a monitor [81]. In this computer-based task, participants are shown either an orange square or a blue square. When the orange square appears, the participant must press the spacebar, but they should withhold any response when the blue square appears. Given the one-second response window, quick execution is required. The task lasted approximately 3 min for 65 trials for each participant. Outcome measures were Reaction Time (RT), and Accuracy (i.e., Percentage of Correct Responses—%CR), which contributed to the appraisal of the Inverse Efficiency Score (IES)—i.e., the division of RT for %CR—across trials. These metrics assessed information processing speed and performance, key in cognitive evaluation.

#### 2.2.5. Mental Effort Measure—Visual Analogue Scale

The Visual Analogue Scale (VAS) has been employed to measure the subjective assessment of perceived difficulty [82], mental fatigue [83], stress and complexity in cognitive tasks [84], and cognitive load [85]. As participants engaged in cognitively demanding tasks assessing attention, working memory, reasoning, and inhibitory control (as previously described), we employed the VAS to measure the adolescents’ mental effort as perceived difficulty both before and after the interventions. Our objective was to determine whether social media use or face-to-face interaction influenced the subjective experience of mental effort. Participants from both the IG and CG were requested to answer the following question: “How difficult did you find these tests?”. Each participant indicated their perceived difficulty by bisecting a 100 mm vertical line, with the position of the mark reflecting how easy or difficult they felt the tests were, providing a subjective measure of the challenge experienced during the tests.

#### 2.2.6. Social Media Usage and Screen Time Measure—Social Media Habits Survey

Excessive screen time and unregulated social media use contribute to cognitive impairments, mental health issues, and overall well-being concerns [86]. Prolonged digital exposure during childhood has been linked to cognitive developmental deficits and adverse socio-emotional outcomes, including diminished attention spans [47]. Therefore, to analyze participants’ behavioral patterns related to social media usage and screen time, we developed a Social Media Habits Survey including four questions applied after the experiment with the adolescents: “How many hours on a weekday do you spend in front of a screen for work?”, “How many hours on a weekday do you spend in front of a screen for leisure?”, “How many hours on a weekend day do you spend in front of a screen for work?”, and “How many hours on a weekend day do you spend in front of a screen for leisure?”. This form aimed to assess whether social media use and screen time outside the intervention influenced its effects, as the IG’s acute exposure lasted only 30 min. Since adolescents likely use social media routinely [87], their overall weekly screen time could contribute to excessive use, potentially impacting cognitive function [6].

As mentioned above, excepting the Social Media Habits Survey, all instruments were applied twice to gather pre-test and post-test measures.

### 2.3. Procedure

**Figure 1:**
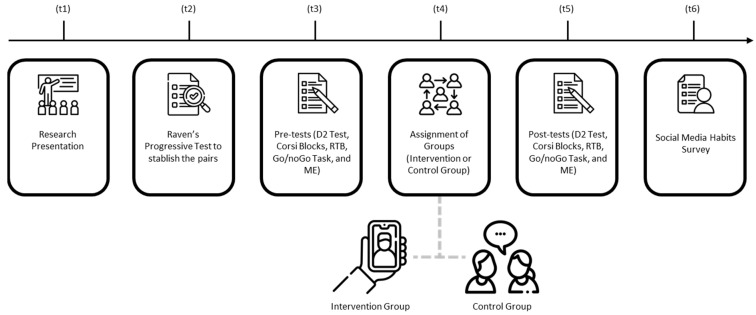
Procedure. Note. RTB = Reasoning Test Battery (BPR); ME = VAS Mental Effort.

The research procedures were structured into five stages, each corresponding to a specific time point (t) as illustrated in Figure 1. After obtaining approval from the school administrations and informed consent and assent, an initial session was held to present the research project to the participants (t1). At the start of the study, the Raven’s Progressive Matrices test [72] was administered during six sessions to 8–15 students per group in a single room (t2). As described before, the aim was to control the potential cofounding effect of the intellectual capacity (G Factor). After, participants were matched according to their G Factor score and their age too, and they were randomly allocated to groups to balance these variables. The interventions were scheduled for later days according to the school agenda. On the scheduled day of the intervention, the pre-tests (D2 Test, Corsi Blocks, BRP, Go/No-Go Task, and VAS) were conducted with each pair of participants in a designated room (t3). Afterward, students moved to adjacent rooms for either social media interaction (IG) or face-to-face conversation (CG) (t4). Following the 30-min interaction period, they returned to the initial room to complete the same tests (t5). Due to school time constraints from the experimental procedure, the Social Media Habits Survey was administered on a separate day, aligning with the school and researchers’ schedules, three weeks later (t6). It is important to note that the same experimenter conducted the study with all participants, minimizing potential bias related to variations in outcome measurements [88]. Additionally, to create a more ecologically valid environment and enhance the generalizability of the results [89], both face-to-face conversations and social media interactions were left unrestricted, allowing participants to engage freely without topic constraints or predefined rules.

### 2.4. Data Analysis

Data analyses were performed using IBM Statistical Package for the Social Sciences (SPSS) v. 29. As mentioned above, through the Social Media Habits Survey, we assessed how many hours a day the participants spent in front of a screen on weekdays, and weekends. After creating a new variable with the total hours per week (hereafter called Screen Time), we verified if the time spent in front of a screen varied between the groups of Interaction Modality (CG—Face-to-Face and IG—Social Media). Results showed statistically significant differences between the interaction modalities (*t*(64) = 2.101, *p* = 0.04, *d* = 0.517). On average, students in the Face-to-Face group spent more time in front of a screen than students in the Social Media group (*M* = 638.64 vs. *M* = 504.42, respectively).

To control the effect of this variable, we tried to apply a Multivariate Analysis of Covariance (MANCOVA) with Screen Time as a covariate, but the assumption of linearity between each dependent variable and the covariate was not met. We found one significant linear relation and a nonlinear pattern in some cases. In such situations, an alternative approach is to recode the continuous variable (in this case, Screen Time) into a categorical variable to use as another factor [90]. To this end, we calculated the median social media usage reported by participants (Me = 540) and divided them into two groups: those spending less time in front of a screen (135 to 540 min per week) and those spending more time (more than 540 to 1320 min per week). So, analyses were performed considering two intergroup factors: Interaction Modality (A), with Face-to-Face interaction group (A_1_) and Social Media interaction group (A_2_); and Screen Time (B), Until 540 min per week (B_1_) and More than 540 min per week (B_2_).

Regarding the ten response variables, we computed the difference between the pre-test and the post-test measures for each variable. Negative scores were eliminated adding the minimum negative value in the variable plus one. Hereafter, these variables will be identified with the following acronyms: RTB_AR (RTB Abstract Reasoning score); D2_TE-S (Total Efficiency score), D2_VI (Variability Index), D2_E% (Error Percentage), and D2_CI (Concentration Index), from D2 test; CORSI_B (Corsi Blocks score, from the instrument with the same name); Go/No-Go RT (Go/No-Go Reaction Time), Go/No-Go_%CR (Go/No-Go Accuracy), and Go/No-Go_IES (Go/No-Go Inverse Efficiency Score), from Go/No-Go Task; and ME (Mental Effort measure, from the Visual Analogue Scale).

Considering that the final experimental design was an A x B factorial design, we tried to perform a Two-Way Multivariate Analysis of Variance (MANOVA). Nevertheless, the scatterplots for each condition (A1B1, A1B2, A2B1, A2B2) showed some non-linear relations or no relation among the dependent variables. Therefore, we applied a Two-Way Analysis of Variance (ANOVA) for each response variable. To control the inflation of Type I error, we used Bonferroni correction of alpha [91]. Since high correlations were observed between the D2_TE-S and D2_CI in the D2 test (r = 0.725, *p* < 0.001), and between the Go/No-Go_IES and Go/No-Go_RT in Go/No-Go Task (r = 0.972, *p* < 0.001), D2_CI and the Go/No-Go_RT were excluded from the analysis. Thus, eight Two-Way ANOVAs were computed, one for each dependent variable (with α = 0.00625). The Two-Way ANOVA for the D2_E% was performed excluding an outlier (z = 6.517).

Concerning the test assumptions, the independence of observations was assumed. Normality was verified for each condition. Most of the dependent variables were normally distributed. However, ANOVA is robust when a violation of normality is not due to outliers [91]. Concerning the homogeneity of the variance across the conditions, the Levene *F* test revealed that the assumption was fulfilled for all the response variables.

## 3. Results

Descriptive statistics for each dependent variable across the conditions are shown in Table 1.

Table 2 shows the results of the Two-Way ANOVAs. We found no statistically significant differences in response variables across Interaction Modality and Screen Time groups, nor a significant interaction between the factors. Therefore, neither Interaction Modality (CG—Face-to-Face and IG—Social Media) nor Screen Time had any impact on any of the eight dependent variables.

## 4. Discussion

This research compares the impact of acute social media exposure on cognitive variables, compared to face-to-face interaction, when screen time per week is from 135 to 540 min and when adolescents spend more than 540 to 1320 min in front of a screen per week. We hypothesized that groups with social media exposure would score lower in attention, working memory, reasoning, and inhibitory control while showing higher mental effort than groups exposed to face-to-face interaction, with stronger effects in those with greater screen time. However, results showed no significant differences in interaction modality, nor any major influence from screen time, indicating that acute social media exposure did not impact these cognitive domains, despite screen time spent per week. So, the results contradict our hypothesis. Two key explanations may account for these results: (i) screen time as a critical factor; and (ii) social media as a cultural ceiling effect.

### 4.1. Screen Time as a Critical Factor

Prolonged use of smartphones has been linked to cognitive and mental health issues in adolescents, with screen time emerging as a key factor influencing these effects [6]. However, our findings suggest that a moderate amount of screen time appears safe, as acute social media exposure showed no detrimental effects. Half of the participants reported weekly usage between 540 and 1320 min (approximately 1 h 20 min to 3 h per day), while the other half reported between 135 and 540 min (20 min to 1 h 20 min per day). Since guidelines advise limiting recreational screen time to two hours per day for individuals aged 5–17, excluding educational activities [92], most of our participants remained within this range (including educational and work-related activities), which may explain the absence of cognitive harm observed in this study. However, the weekly screen time observed in our sample does not reflect the reality for most adolescents today.

Adolescents aged 17–18 spend an average of six hours daily on screens [93], and a large-scale study of 14.9-year-olds recorded a 129-min increase in daily screen time between 2019 and 2022, rising from 320 min (5.3 h) to 449 min (7.5 h), nearly matching sleep duration in 2022 [94]. While our findings indicate that keeping screen time near the recommended two recreational hours daily may not be harmful, this scenario may not be realistic worldwide. In this way, screen time duration is a key factor in determining its cognitive impact.

Prolonged screen time among adolescents is associated with poorer mental health and sleep quality [95] and problematic smartphone and social networking use [96]. Excessive screen time during the critical stages of childhood and adolescence may pose risks to cognitive development and brain health [97]. Adolescent girls generally spend more screen time on social media than boys, who tend to engage more with video games [98], which can also lead to health issues associated with excessive screen time [99]. However, social media usage is common among both genders, with negligent behaviors, such as sharing personal information on their accounts, being more closely linked to age than to gender (i.e., minors are more negligent) [100]. Excessive social media and internet use can provoke extreme emotions among adolescents, lower self-esteem through social comparison, and contribute to mental health issues and decreased academic performance in adolescents [101]. Social media influences adolescents’ behavior, and neurobiology, amplifying developmental changes that heighten vulnerability to mental health issues through mechanisms such as risky self-presentation, social comparison, sensitivity to social feedback, exclusion experiences, and altered stress and reward processing [102].

Nonetheless, if our results suggest that moderate social media use does not harm cognition, it may even offer benefits beyond cognitive function. Engaging with peers and sharing content can enhance social support networks, improve self-esteem, and reduce loneliness [103]. Social media also serves as a tool for entertainment, education, and self-improvement, providing access to various learning formats such as videos, audiobooks, reviews, and lectures [104]. It seems like when screen time is kept within moderate limits, social media can offer benefits beyond cognition without adverse effects. If screen time is key to determine its benefits or risks, it is necessary to think about digital literacy and adopt a holistic approach involving the contexts surrounding the youth, such as family, schools, and government. Digital literacy extends beyond basic internet navigation and social media use, requiring education on technology management, opportunities, and risks [105]. Educating parents and teachers is essential, and teaching adults to handle technology effectively can foster healthier digital habits in children [106]. Parents and educators should understand digital tools’ potential and limitations, integrating them into learning while setting a positive example, as their screen habits influence child development [107]. Concerning broader aspects, driven by government policies and sociopolitical, cultural, and economic factors, integrating digital tools into schools has become a growing trend, reshaping education [108]. However, some countries, like France and China, have taken the opposite approach, restricting smartphone use in schools [109]. Therefore, while the impact of screens on children is an important issue, broader social awareness and alignment are equally vital. Digital literacy should include adults, considering socioeconomic, cultural, and family factors before regulating or stimulating technology in schools. Addressing early digital exposure and excessive screen time requires a holistic approach among educators, policymakers, and parents for balanced integration, and avoiding excessive use [110]. Just as laws and awareness campaigns targeting youth address risky behaviors like alcohol, drugs, and sexual health—topics that significantly impact adolescents [111]—social media should be approached with the same level of urgency.

### 4.2. Social Media as a Cultural Ceiling Effect

Adolescents have incorporated social media use into their daily routines [112]. Our findings indicate that an extra 30 min of social media use may not have significantly influenced cognition, potentially due to social media’s deep integration into modern digital culture. During childhood and adolescence, socialization is pivotal in developing cultural identity by embedding societal norms and values [113]. Social media, facilitated by information and communication technologies (ICT) [114], influences this socialization process by creating virtual spaces where individuals interact for work, education, leisure, and communication [115,116]. As a result, individual behaviors are influenced by cultural factors such as nationality, region, and ethnicity, as well as by the ICT tools they engage with [117,118]. While cultural differences influence how people interact with social media, reflecting regional distinctions [119] and diverse shared global values and lifestyles [120], social media has emerged as a common interest. Social media’s widespread presence transcends these differences, embedding itself in daily life and fostering a global modern digital culture that bridges diverse societies through universal platforms [121,122]. A 2022 global study reported that there were 4.62 billion social media users worldwide, with an average daily usage time of 2 h and 27 min [123], highlighting the pervasive role of social media in modern global life.

In the United States, studies on adolescents aged 13–19 reveal that 90% have access to social media platforms, with over half spending more than four hours daily on these platforms [124]. Social media plays a dominant role in shaping the cultural identity of recent generations, driving significant changes in language use and social values, while simultaneously acting as a platform for both cultural preservation through online communities and cultural erosion due to the pervasive influence of foreign cultures [125]. Social media use among youth has become a habitual activity for social connection, identity exploration, self-expression, informational and social support, and access to information about general content, news, and events [126].

Social media, as an integral part of digital culture for the youth, may contribute to a habitual usage effect, where brief interactions neither enhance nor diminish cognitive performance. In social and behavioral sciences, when a task is too simple to challenge participants, a phenomenon known as the ceiling effect often occurs, leading to uniformly high performance [127]. Similarly, in this study, the absence of cognitive differences following the additional 30 min of social media use could be attributed to participants’ familiarity with social media as a regular aspect of their daily lives. This cultural integration likely made the stimulus insufficient to create any measurable cognitive variation, resembling the dynamics observed in a ceiling effect.

However, retaking a previously cited study, merely having a phone nearby taxed attentional resources tied to fluid intelligence and working memory [14]. Comparing this with our findings raises an alarming possibility: the cultural integration of screen use may resemble addictive behavior. Digital technology’s addictive nature may stem from dopamine-driven learning and motivation, with habitual activity stimulating this neurotransmitter [128]. Repeated behaviors can form addictive habits [129], like daily screen use. Since all our participants had daily screen exposure, this habitual use may explain both studies. Smartphone use can follow addiction-like patterns, including withdrawal symptoms, compulsive use to satisfy cravings, and problematic behaviors [130]. Addictive smartphone use involves dependence to regulate mood, agitation when deprived, and failed attempts to reduce usage [131]. Therefore, in the first study [14], participants’ phone absence may have triggered agitation, whereas in our study, allowing screen use may have satisfied this habitual need, preventing immediate cognitive impact.

Our results indicate that acute social media use might not affect youth cognition. However, the normalization of excessive screen time as a cultural component can be problematic as it resembles addictive behavior. Social media platforms compete for attention, and, going a little bit further, they are leveraging artificial intelligence (AI)-driven algorithms to personalize content, maximize engagement, and intensify reward center activation—creating a feedback loop that fosters addictive behaviors and raises ethical concerns about privacy and digital personalization [132]. Without awareness and regulation, unchecked exposure to constant digital stimuli just as something normal could lead to a generation increasingly vulnerable to cognitive overload and addiction in an environment designed to keep them hooked. Educators and researchers must lead discussions across institutions to regulate and educate youth about social media use, while companies should take greater responsibility in marketing strategies and product development—rather than exploiting neurophysiological brain insights to compete for consumer attention [133].

## 5. Conclusions

This study examined acute social media exposure versus face-to-face interaction on adolescent cognition, related to attention, working memory, reasoning, inhibitory control, and mental effort, considering a habit of less or more time per week in front of a screen. Contrary to our initial hypothesis, no significant differences emerged, suggesting brief social media use does not impair cognitive performance, despite screen time spent per week. Two key factors may explain this: screen time and social media as a cultural norm. While excessive screen time can affect cognition and mental health, our findings support that social media interaction under the recommended two-hour daily limit [92] may not negatively affect cognitive function, as most participants stayed within this range. Social media’s deep integration into adolescent life may create a “cultural ceiling effect”, where brief use has no measurable impact. As a central feature of modern digital culture, its familiarity may neutralize the cognitive effects of acute exposure. However, habitual screen exposure can mirror addiction, reinforcing compulsive use and withdrawal symptoms. Digital literacy efforts involving families, schools, governments, and the companies behind social media are essential.

## 6. Limitations and Future Research

We acknowledge several limitations in this study, starting with the duration of social media exposure during the experiment. Due to time constraints within school schedules and participants’ commitments, the intervention was limited to a brief exposure period. It is essential to investigate whether longer exposure, particularly beyond the recommended two recreational daily hours for adolescents [92], would have different cognitive impacts. Also, while we assessed participants’ weekly screen time, our discussion focused only on the 30 min of daily use during the study. However, we did not evaluate if participants had already accessed screens earlier that day, potentially accumulating more daily screen time. Future research should control and explore longer exposure durations, emphasizing the importance of adhering to health guidelines to mitigate the negative effects of prolonged screen time and social media use.

Since this study focused on adolescents of similar ages from two schools in Portugal, its findings may reflect a narrow group with shared characteristics, potentially limiting applicability to general adolescents. Additionally, Social media use is more common among girls than boys [98], and younger adolescents may exhibit more negligent behavior on social media compared to older ones [100]. Our study did not examine gender or age differences, as both genders were combined for a more homogeneous sample, and age variation was minimal. Future research should explore these variables to assess whether social media affects boys and girls or different age groups differently. We hypothesize that girls may experience a stronger “cultural ceiling effect” due to habitual use or greater vulnerability to negative cognitive impacts from addictive behaviors. Similarly, younger adolescents may be more susceptible to cognitive impacts due to their higher levels of negligent social media use.

Future research should consider randomized controlled trials to complement our findings and further examine the impact of acute social media exposure on cognition. Additionally, this study examined only four cognitive domains (i.e., attention, working memory, reasoning, and inhibitory control), but social media may also influence other cognitive functions, such as learning [134], language [135], and decision-making [136], which warrant further investigation. Given the intense emotions social media often triggers in adolescents [101], future research should also focus on its emotional impacts and its role in shaping youth cognition.

Our study was conducted in an ecological setting (i.e., schools), enhancing external validity by allowing findings to be more easily generalized to real-life contexts [89]. However, this setting limits the use of neurophysiological tools like EEG or Functional magnetic resonance imaging (fMRI), which provide objective cognitive measurements in assessing social media’s impact on the brain [137,138]. Additionally, the self-reported survey on social media habits introduces potential bias due to the subjective nature of the responses. A more accurate approach to measuring social media usage would be to track screen time directly within the social media applications. However, this raises ethical and privacy concerns that must be carefully considered in research. Future studies should integrate neurophysiological instruments to assess neural activity during acute social media exposure and explore objective methods for measuring screen time. These approaches would provide deeper insights into the cognitive effects of social media use.

Lastly, the relationship between social media use and adolescent psychosocial outcomes varies in the literature, urging researchers to move beyond screen time and investigate how specific content, features, and functions interact with individual traits and offline experiences to influence development [139]. Despite growing research, consensus on social media’s impact on adolescent well-being remains limited, largely due to the focus on usage frequency and specific well-being indicators, highlighting the need for a broader discussion that considers contextual factors, varied indicators, and both positive and negative effects [140]. Future research should explore both quantitative and qualitative aspects of social media beyond screen time to foster a more comprehensive discussion on the topic.

## Figures and Tables

**Table 1:** Descriptive statistics for each response variable across the conditions.

Interaction Modality	Screen Time		*M*	*SD*	*N*
CG—Face-to-Face	Until 540 min	RTB_AR	7.13	3.523	15
		D2_TE-S	159.87	33.579	15
		D2_VI	15.27	6.497	15
		D2_E%	17.28	2.804	15
		CORSI_B Go/No-Go_%CR	46.67 1.07	16.663 0.019	15 15
		Go/No-Go_IES ME	144.71 35.07	43.601 21.740	15 15
	More than 540 min	RTB_AR	6.56	2.148	18
		D2_TE-S	156.33	34.152	18
		D2_VI	17.78	5.579	18
		D2_E%	18.38	1.467	18
		CORSI_B Go/No-Go_%CR	43.39 1.06	12.448 0.017	18 18
		Go/No-Go_IES ME	156.96 28.28	43.262 15.025	18 18
IG—Social Media	Until 540 min	RTB_AR	6.53	2.091	19
		D2_TE-S	151.05	45.980	19
		D2_VI	18.11	5.685	19
		D2_E%	18.19	1.703	19
		CORSI_B Go/No-Go_%CR	44.95 1.07	16.622 0.031	19 19
		Go/No-Go_IES ME	111.02 30.42	56.596 12.611	19 19
	More than 540 min	RTB_AR	7.14	2.905	14
		D2_TE-S	152.00	40.633	14
		D2_VI	16.00	9.240	14
		D2_E%	21.625	8.009	13
		CORSI_B Go/No-Go_%CR	53.36 1.05	16.003 0.024	14 14
		Go/No-Go_IES ME	149.55 35.14	50.114 13.581	14 14

Note. RTB_AR = RTB Abstract Reasoning; D2_TE-S = D2 Total Efficiency Score; D2_VI = D2 Variability Index; D2_E% = D2 Error Percentage; CORSI_B = Corsi Blocks score; Go/No-Go_%CR = Go/No-Go Accuracy; Go/NoGo_IES = Go/No-Go Inverse Efficiency Score; ME = VAS Mental Effort.

**Table 2:** Two-Way ANOVA for each dependent variable through the conditions.

Variable	Sources	Hypoth. Sum of Squares	df, df	Hypoth Mean Square	F	Sig.	Partial η^2^
RTB_AR	Interaction Modality Screen Time Interaction Modality*Screen Time	0.002 0.006 5.792	1, 62 1, 62 1, 62	0.002 0.006 5.792	0.000 0.001 0.815	0.988 0.977 0.370	0.000 0.000 0.013
D2_TE-S	Interaction Modality Screen Time Interaction Modality*Screen Time	701.850 27.153 81.519	1, 62 1, 62 1, 62	701.850 27.153 81.519	0.457 0.018 0.053	0.501 0.895 0.818	0.007 0.000 0.001
D2_VI	Interaction Modality Screen Time Interaction Modality*Screen Time	4.569 0.669 86.530	1, 62 1, 62 1, 62	4.569 0.669 86.530	0.101 0.015 1.908	0.752 0.904 0.172	0.002 0.000 0.030
D2_E%	Interaction Modality Screen Time Interaction Modality*Screen Time	4.092 7.720 2.594	1, 61 1, 61 1, 61	4.092 7.720 2.594	0.258 0.486 0.163	0.614 0.488 0.688	0.004 0.008 0.003
CORSI_B	Interaction Modality Screen Time Interaction Modality*Screen Time	276.290 106.940 554.643	1, 62 1, 62 1, 62	276.290 106.940 554.643	1.156 0.447 2.320	0.287 0.506 0.133	0.018 0.007 0.036
Go/No-Go_%CR	Interaction Modality Screen Time Interaction Modality*Screen Time	3.240 × 10^−6^ 0.002 0.000	1, 62 1, 62 1, 62	3.240 × 10^−6^ 0.002 0.000	0.006 3.513 0.182	0.940 0.066 0.671	0.000 0.054 0.003
Go/No-Go_IES	Interaction Modality Screen Time Interaction Modality*Screen Time	6860.091 10,470.192 2803.239	1, 62 1, 62 1, 62	6860.091 10,470.192 2803.239	2.860 4.364 1.169	0.096 0.041 0.284	0.044 0.066 0.018
ME	Interaction Modality Screen Time Interaction Modality*Screen Time	20.002 17.349 537.984	1, 62 1, 62 1, 62	20.002 17.349 537.984	0.079 0.068 2.123	0.780 0.794 0.150	0.001 0.001 0.033

Note. Bonferroni correction (*α* = 0.00625). RTB_AR = RTB Abstract Reasoning; D2_TE-S = D2 Total Efficiency Score; D2_VI = D2 Variability Index; D2_E% = D2 Error Percentage; CORSI_B = Corsi Blocks score; Go/No-Go_%CR = Go/No-Go Accuracy; Go/NoGo_IES = Go/No-Go Inverse Efficiency Score; ME = VAS Mental Effort.

## Data Availability

The data presented in this study is freely available at the OSF repository at https://osf.io/cnr84/?view_only=cc0c4dd052bc41b58e8641815e7578d5 with the following DOI: https://www.doi.org/10.17605/OSF.IO/CNR84.
